# A fermentation process for the production of poly(3-hydroxybutyrate) using waste cooking oil or waste fish oil as inexpensive carbon substrate

**DOI:** 10.1016/j.btre.2022.e00700

**Published:** 2022-01-11

**Authors:** Tran Thi Loan, Dao Thi Quynh Trang, Pham Quang Huy, Pham Xuan Ninh, Doan Van Thuoc

**Affiliations:** a Department of Biotechnology and Microbiology, Faculty of Biology, Hanoi National University of Educaction, 136 Xuan Thuy, Cau Giay, Hanoi, Vietnam; b Department of Microbiology, Faculty of Biology, University of Science, Vietnam National University, Hanoi (VNU), 334 Nguyen Trai, Thanh Xuan, Hanoi, Vietnam; c Ngo Quyen High School, 2 Me Linh, Le Chan, Hai Phong, Vietnam; d Tran Nguyen Han High School, 186 Ton Duc Thang, Le Chan, Hai Phong, Vietnam

**Keywords:** Cupriavidus necator, Poly(3-hydroxybutyrate), Polyhydroxyalkanoate, Waste cooking oil, Waste fish oil

## Abstract

The utilization of waste cooking oil (WCO) or waste fish oil (WFO) as inexpensive carbon substrate for the production of poly(3-hydroxybutyrate) (PHB) by *Cupriavidus necator* H16 was investigated. Fed-batch cultivation mode in bioreactor was applied in this study. High cell dry weight (CDW) of 135.1 g/L, PHB content of 76.9 wt%, PHB productivity of 1.73 g/L/h, and PHB yield of 0.8 g/g were obtained from WCO. In the case of WFO, the CDW, PHB content, PHB productivity, and PHB yield were 114.8 g/L, 72.5 wt%, 1.73 g/L/h, and 0.92 g/g, respectively. The PHB productivity and yield obtained in the current study from WCO or WFO are among the highest reported so far for PHA production using oils as sole carbon substrate, suggesting that both WCO and WFO can be used as inexpensive carbon substrates for the production of PHA on an industrial scale.

## Highlights

• Both WCO and WFO can be used as promising substrates for PHA production.
• First report of a fed-batch fermentation process using WFO as sole carbon source for PHA production.
• High PHB yields of 0.8 g/g and 0.92 g/g were produced from WCO and WFO, respectively.
• Highest PHB productivity (1.73 g/L/h) was achieved when using waste oil as carbon source.

## Graphical abstract

**Figure:**
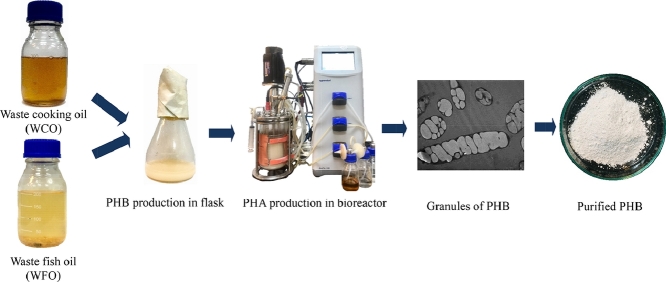

## Table of Abbreviations

^1^H NMR: Proton nuclear magnetic resonance
C/N: Carbon/nitrogen
CDW: Cell dry weight
mcl: Medium chain length
OD: Optical density
P(3HB-*co*-3HH_X_): Poly(3-hydroxybutyrate-*co*-3-hydroxyhexanoate)
P(3HB-*co*-3HO): Poly(3-hydroxybutyrate-*co*-3-hydroxyoctanoate)
PHB: Poly(3-hydroxybutyrate)
PHA: Polyhydroxyalkanoate
PM: Production medium
SM: Seed medium
WCO: Waste cooking oil
WFO: Waste fish oil
wt%: Percent of cell dry weight

## 1 Introduction

Polyhydroxyalkanoates (PHAs) are a group of biodegradable polymers synthesized by many bacteria and archaea as carbon and energy-storage materials [[1], [2], [3], [4], [5]]. So far, there are more than 150 PHA monomer subunits that have been identified, and among them, poly(3-hydroxybutyrate) (PHB) is a common biopolymer found in nature [1, 6]. After purification, PHAs display some useful properties such as biodegradable, biocompatible, and recyclable plastics. These properties render them highly competitive with some petrochemical-based synthetic plastics currently in use for making disposable items such as bottles, containers, cups, and bags [7], [8], [9], as well as medical materials such as heart valves, vessel stents, sutures, and skin substitutes [9, 10]. However, the wide applications of PHAs are hindered by their high production cost. The price of the carbon substrate used in the PHA production process is a major reason, contributing up to 50% of the total production cost [11, 12]. Hence, the utilization of inexpensive and renewable carbon substrates such as agricultural or industrial residues is offering a main solution for reducing PHA production cost [13], [14], [15], [16], [17], [18], [19], [20].

The carbon substrates for biosynthesizing PHAs include carbohydrates, hydrocarbons, and triacylglycerol (Table 1). Among the three kinds of carbon substrates used for PHA production, the productivity of PHAs from carbohydrates and hydrocarbons are normally lower as compared with triacylglycerol. The yields of PHAs from glucose and sucrose, two common carbohydrates for PHA production, are approximately 0.33–0.48 g/g. On the other hand, triacylglycerols such as plant oils have been shown to provide the highest yields, up to 0.72–0.83 g/g (Table 1). For this reason, most recent studies have been focused on the use of triacylglycerol from different origins as carbon substrates for biotechnological PHA production [31], [32], [33], [34], [35], [36], [37], [38], [39], [40], [41], [42]. Waste plant oils are considered as inexpensive and excellent substitute carbon substrates for commonly used in PHA production [6]. Some bacteria such as *Cupriavidus necator* [31], [32], [33], *Pseudomonas* [39], [40], [41], [42], and *Bacillus* [42] species can efficiency convert waste plant oil into PHA. Among them, *C. necator* always emerged as the best PHA-producing bacterial species. The highest cell dry weight (CDW) of 138 g/L, PHA concentration of 105 g/L, and PHB productivity of 1.46 g/L/h were achieved by the *C. necator* H16 strain after 72 h of cultivation using waste rapeseed oil and propanol as carbon sources [32].

**Table 1:** Comparison of PHA production from different carbon substrates by some high producing strains.

Organism	Carbon source	PHA type	CDW (g/L)	PHA content (wt%)	PHA conc. (g/L)	PHA productivity (g/L/h)	PHA yield (g/g)	Reference
*Protomonas extorquens*	Methanol	PHB	233	64	149	0.88	0.2	Suzuki et al. [21]
*Methylobacterium organophilum*	Methanol	PHB	250	52	130	1.86	0.19	Kim et al. [22]
*Pseudomonas oleovorans*	n-Octane	P(3HHx-*co*-3HO)^a^	37.1	33	12.1	0.25	–	Preusting et al. [23]
*C. necator*	Glucose	PHB	164	73.8	121	2.42	0.33–0.48	Kim et al. [24]
*Halomonas* TD01	Glucose	PHB	83	78	64.7	1.34	0.34	Tan et al. [25]
*Alcaligenes latus*	Sucrose	PHB	143	50	71.5	3.97	0.4	Yamane et al. [26]
*Alcaligenes latus*	Sucrose	PHB	112	88	98.7	4.94	0.42	Wang and Lee [27]
*Bacillus megaterium*	Sugarcane molasses	PHB	72.6	42.1	30.5	1.27	0.07	Kulpreecha et al. [28]
*Zobellella denitrificans*	Glycerol	PHB	82.1	66.9	54.3	1.1	0.25	Ibrahim and Steinbuchel [29]
*Burkholderia sacchari*	Wheat straw hydrolysate	PHB	145.8	72	105	1.72	0.22	Cesario et al. [30]
*C. necator*	Plant oils	PHB	120	62.5		0.96	0.72–0.76	Kahar et al. [31]
*C. necator*	Waste rapeseed oil	PHB	138	76	105	1.46	0.83	Obruca et al. [32]

a Poly(3-hydroxybutyrate-*co*-3-hydroxyoctanoate).

In addition, the use of animal oils for PHA production has also been studied. The bioconversion of crude fish oil into PHA was first reported by Ashby and Solaiman [41]. They found six strains of *Pseudomonas* that can convert hydrolyzed pollock oil into PHA with polymer contents ranging from 6 to 53 wt% of the CDW. The production of PHB by *Ralstonia* sp. M91 using crude fish oil as carbon substrate was also investigated. In a flask experiment, maximum CDW and PHB concentration obtained by strain M91 were 3.93 g/L and 2.43 g/L, respectively, when the culture medium was supplied with 15 g/L crude fish oil [35]. A high bacterial cell mas of 10 g/L and PHB concentration of 5.2 g/L were obtained by *Salinivibrio* sp. strain M318 after 48 h of cultivation in a flask experiment using mixtures of waste fish oil and glycerol as carbon sources, as well as fish sauce as nitrogen source. By the use of fed-batch cultivation mode, a CDW of up to 69.1 g/L and a PHB concentration of 35.6 g/L were achieved by this halophilic bacterial strain after 78 h of cultivation [36].

However, as shown in the Table 1 and mentioned in a previous report by Jiang et al. [13], the yields of PHAs from oils is high, but the productivity of PHAs using oils is still lower than that using carbohydrates. In order to improve the PHA productivity from waste oils, we carried out this study with the aim of developing an efficient fermentation process using cheap and available waste oils, including waste fish oil (WFO) and waste cooking oil (WCO) as carbon substrates for PHA production by the bacterial strain *C. necator* H16. The use of the low-cost substrates, combined with the bacterium's efficient PHA production capability, could lead to the production of relatively cheap PHA. This study will help to reduce biopolymer production cost, add value to waste oils, as well as reduce environmental pollution caused by waste oils.

## 2 Materials and methods

### 2.1 Bacterial strain and maintenance

*C. necator* H16 (DSM 428) was grown on solid medium containing the following (g/L): meat extract, 10; peptone, 10; yeast extract, 2; granulated agar, 20; and pH 7.0. The culture was maintained at 4 °C and transferred monthly.

### 2.2 Samples

WFO from Basa fish (*Pangasius bocourti*) was collected from Ho Chi Minh City (Vietnam) and used for this study. The major fatty acids were oleic acid (38.6%), palmitic acid (30.6%), linoleic acid (9%), stearic acid (8.2%), myristic acid (4.2%), and palmitoleic acid (2.5%) [35].

WCO was derived from soybean oil, which was provided by a restaurant in Hai Phong City (Vietnam). The main fatty acid composition of the soybean oil and WCO was reported in previous studies [43], [44], [45]: linoleic acid (∼55%), oleic acid (20%–22%), palmitic acid (10%–12%), linolenic acid (5%–8%), and stearic acid (∼4%).

### 2.3 PHA production in shake flasks

The bacterial strain *C. necator* H16 was first grown in seed medium (SM) containing the following (g/L): meat extract, 10; peptone, 10; and yeast extract, 2. This was done in a rotary shaker incubator at 30 °C and 180 rpm for 15 h. Subsequently, the seed culture broth was inoculated at a concentration of 5% (v/v) into 250 mL Erlenmeyer flasks containing 50 mL of medium for PHA production (PM1) (Table 2). In this experiment, MP1 consisted of 10, 15, 20, 25, or 30 g/L of WCO or WFO. The pH of the medium was initially adjusted to 7.0. The cultures were incubated at 30 °C with rotary shaking at 180 rpm. After 48 h of growth, the samples were harvested by centrifuging for CDW and PHA analysis.

**Table 2:** Medium composition for PHA production by *C. necator* H16 in batch and fed-batch fermentation.

Component	PM1 medium		PM2 medium	
	Batch (g)^b^	Feed (g)^b^	Batch (g)^b^	Feed (g)^c^
Carbon source	20	V	20	V
NaH_2_PO_4_∙2H_2_O	5	5	5	–
Na_2_HPO_4_∙12H_2_O	11.6	11.6	11.6	–
Urea	0.54	5.4	2.2	440
MgSO_4_∙7H_2_O	0.39	3.9	0.39	390
K_2_SO_4_	0.45	4.5	0.45	150
CaCl_2_∙2H_2_O	0.06	0.6	0.06	60
Peptone	1	10	–	
Meat extract	1	10	–	
Yeast extract	0.4	4	–	
Trace elements^a^	–	–	1 mL	1 mL

V: Component concentration was varied in different experiments.

a Trace elements contain the following (g/L): CuSO_4_∙5H_2_O, 0.48; ZnSO_4_∙7H_2_O, 2; MnSO_4_∙H_2_O, 2.4; FeSO_4_∙7H_2_O, 15; and 1 L of 0.1 N HCl.

b All components were dissolved in 1 L water except the carbon source.

c Each component was prepared separately.

### 2.4 PHA production in batch fermentation

*C. necator* H16 was initially grown in three different 250 mL flasks containing 50 mL of SM for 15 h at 30 °C with rotary shaking at 180 rpm. The seed culture was then used to inoculate 1.35 L of MP1 containing 2% (w/v) WCO or WFO as carbon source in a 3 L bioreactor (Eppendorf BioFlo 120). Batch fermentation mode was used out at 30 °C, antifoam was added when needed, and the pH of the culture medium was maintained at 7.0 by adding 3 M H_3_PO_4_/NaOH. The agitation speed and air inflow rate were initially set at 200 rpm and 0.5 L/min and were increased during the fermentation to 500 rpm and 1.5 L/min, respectively. Samples were taken every 6 h for optical density (OD600), CDW, and PHA analyses.

### 2.5 Fed-batch fermentation for PHA production

Two culture media (PM1 and PM2) were used in the fed-batch fermentation process (Table 2). *C. necator* H16 was initially grown in a 100 mL flask containing 25 mL of SM for 15 h at 30 °C with rotary shaking at 180 rpm. The culture was inoculated into three different 250 mL flasks containing 50 mL of PM1 or PM2 at a concentration of 5% (v/v). After 15 h of cultivation, 150 mL of the PM1 or PM2 cultures were used to inoculate a 3 L bioreactor vessel containing 1.35 L of fermentation medium (PM1 or PM2). Stirring velocity and aeration, initially set at 200 rpm and 0.5 L/min, respectively, were increased during the fermentation to 800 rpm and 1.5 L/min, respectively. The temperature was set at 30 °C, and pH was maintained at 7.0 by adding 3 M H_3_PO_4_/NaOH. Antifoam was added to the bioreactor when needed.

For the fermentation process using PM1, 25 mL of the feed solution containing 10 × concentrated PM1 was pumped into the bioreactor every 3 h, starting at 12 h until 36 h of cultivation. For the fermentation process using PM2, different feed solutions were pumped into the bioreactor, starting at 12 h until 48 h of cultivation. The stock solution of 44% urea was pumped into the bioreactor every 6 h at a concentration of 5 mL/L; trace element solution, 15% K_2_SO_4_, 39% MgSO_4_∙7H_2_O, and 6% CaCl_2_∙2H_2_O were also pumped into the bioreactor every 12 h at concentrations of 1, 3, 1, and 1 mL/L, respectively.

After 6 h of cultivation, the carbon source (WCO or CFO) was added to the bioreactor every 3 h based on the increase in OD600 value during the growth phase (OD600 = 3 is equivalent to 1 g/L carbon substrate). The total WCO and WFO used in fed-batch fermentation were 160 g/L and 120 g/L, respectively. Samples were taken every 3 h for OD600 analysis and every 6 h for CDW and PHA analyses.

### 2.6 Analytical methods

Transmission electron microscopy (JEM-1010; Jeol Ltd., Tokyo, Japan) was performed for the observation of PHA granules in the bacterial cells [46].

OD600 was determined by centrifuging 1 mL of the culture samples at 5 000 *g* for 10 min in centrifuge tubes. The pellet was washed once with hexane and once with distilled water and diluted with distilled water, and then the absorbance was read at 600 nm.

CDW was determined by centrifuging 3 mL of the culture samples at 5 000 *g* for 10 min in centrifuge tubes. The pellet was washed once with hexane and once with distilled water, centrifuged, and dried at 105 °C until constant weight was obtained. The centrifuge tube was weighed again to calculate the CDW [36].

The residual oil concentration in the culture broth was determined using a method described by Kahar et al. [31]. The structure of the accumulated polymer in the bacterial cells was determined by ^1^H NMR method as described by Thuoc et al. [36]. PHA quantification was performed using a gas chromatographic method described by previous reports [35, 47]. Poly(3-hydroxybutyrate-*co*-3-hydroxyvalerate) containing 12% valerate (Sigma) was used as a reference for the peak identification.

PHB content (weight percent, wt%) was calculated as the percentage of the ratio of PHB concentration to CDW. PHB yield was calculated as concentration of synthesized PHB divided by the amount of carbon substrate consumed [48].

## 3 Results and discussion

### 3.1 PHA synthesis from different concentrations of waste oil

**Fig. 1:**
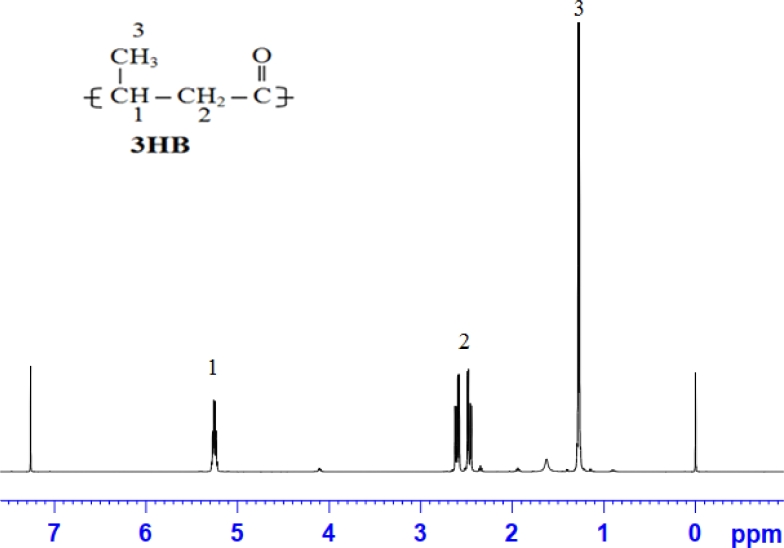
^1^H NMR spectra of PHA extracts from *C. necator* H16 grown on WCO.

**Fig. 2:**
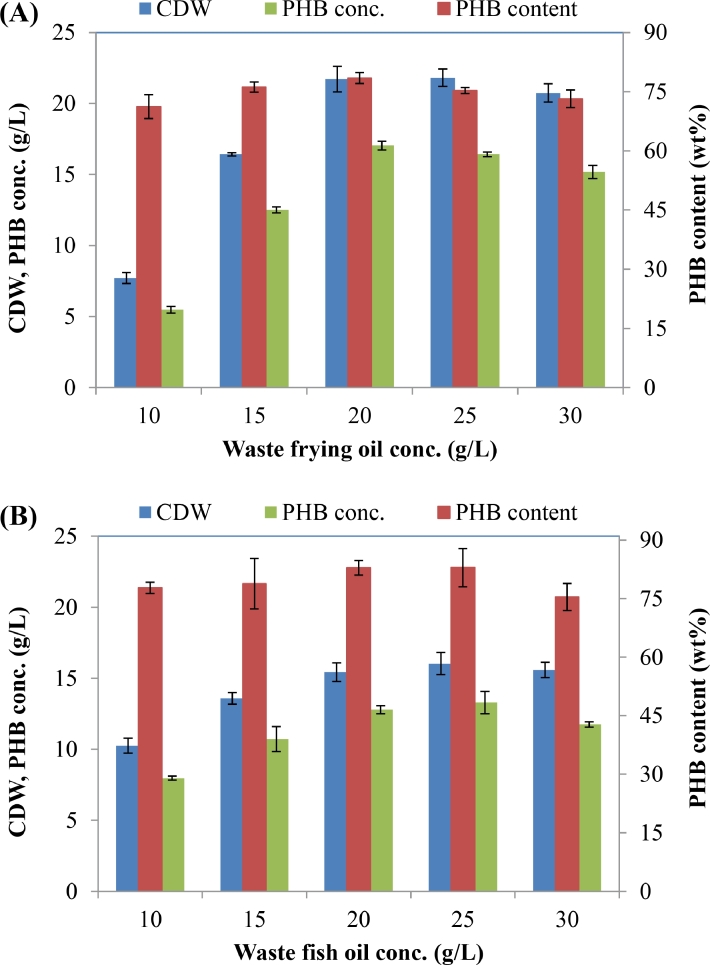
Effect of different concentrations of WCO (A) and WFO (B) on cell mass concentration and PHA accumulation by *C. necator* H16.

**Fig. 3:**
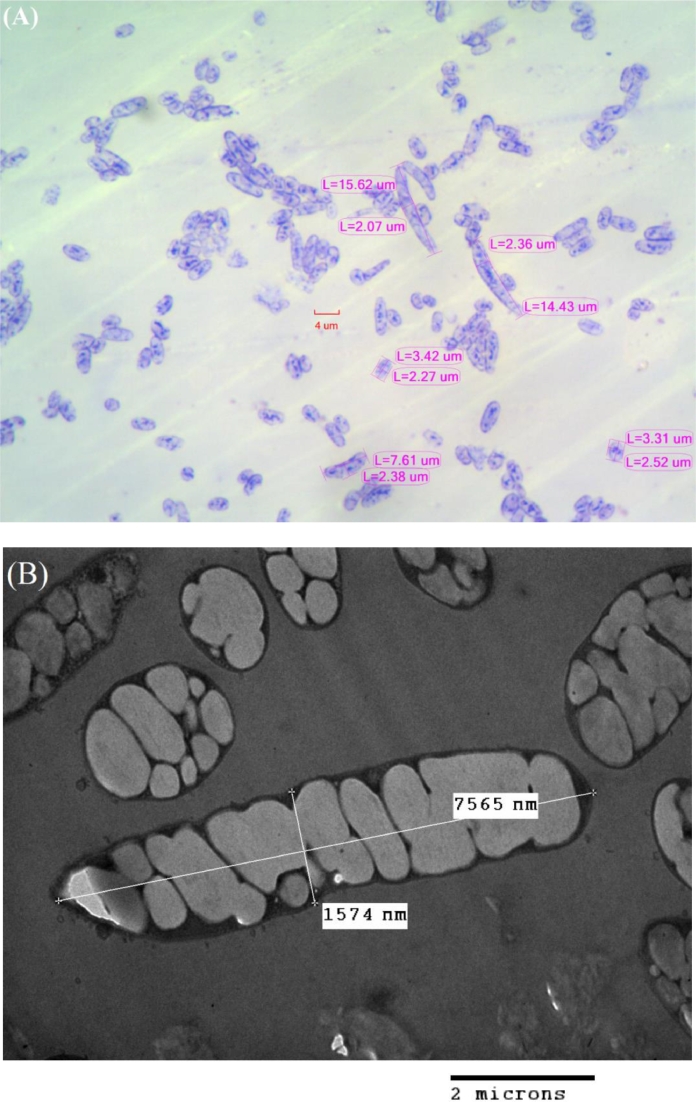
Light microscopy image showing the morphology of bacterial cells (A) and transmission electron microscopy image showing the morphology of PHB granules and bacterial cells (B) using 20 g/L WFO as carbon substrate after 48 h of cultivation.

The effect of different concentrations of WCO or WFO on the growth rate of and PHA production by *C. necator* H16 was evaluated. Samples were taken and analyzed after 48 h of cultivation. The results of ^1^H NMR analysis showed that *C. necator* H16 strain synthesized homopolymer PHB from WCO or WFO (Fig. 1). High CDW and PHB contents were obtained when the medium was supplemented with waste oil at concentrations between 20 and 25 g/L. Maximum PHB contents of 78.5 and 82.9 wt% were accumulated by *C. necator* H16 when 20 g/L of WCO and WFO were supplied, respectively (Fig. 2). Maximum PHB concentrations of 17 g/L and PHB yield of 0.85 g/g were obtained from 20 g/L of WCO (Fig. 2A). In the case of using WFO, the highest PHB concentration of 13.3 g/L and PHB yield of 0.8 g/g were achieved when 25 and 10 g/L, respectively, of WFO were supplied (Fig.  2B). The results indicate that both WCO and WFO can be considered as promising carbon substrates for PHB production by *C. necator* H16. Interestingly, as can be seen from Fig. 3, the PHB granules filled the bacterial cells, and the bacterial cells became bigger when the content of accumulated PHB was high. Normally, the size of bacterial cells was only 0.8–1.2 × 1.5–2.5 µm, but after 48 h of cultivation in the medium containing waste oil as carbon substrate, the bacterial size increased to 1.5–2.5 × 3.0–15 µm (Fig. 3A). This unusual bacterial size caused by high PHB content suggests that the provided waste oil was mainly used for PHB accumulation.

There are few studies on PHA production by *C. necator* using WCO as carbon substrate. Waste frying oil (rapeseed oil) was used as carbon source for PHB production by *C. necator* H16, and PHB concentration of 1.2 g/L was achieved after 48 h of cultivation in a medium containing 20 g/L oil [49]. Another study also reported that the *C. necator* strain was able to convert different kinds of waste oils into PHB. High PHB concentrations of 5.8, 7.7, 6.7, and 6.8 g/L were obtained when waste sunflower oil (household), waste rapeseed oil (university canteen), waste sunflower oil (restaurant), and waste rapeseed oil (chips manufactory) were respectively used as carbon source [32]. The highest PHB concentration of 18 g/L was obtained with *C. necator* using waste palm oil [50]; this is in the same range obtained in this study using waste soybean oil (PHB concentration of 17 g/L).

Maddikeri et al. [51] estimated that the amount of WCO generated each year in the globe is about 29 million tons. In many countries, the collected WCO is mainly used for the production of biodiesel. However, the concentration of free fatty acids in WCO is normally high and reduces the yield of conversion rate. Therefore, a pretreatment step is often conducted to reduce the concentration of free fatty acids [52, 53]. In contrast, WCO can be directly converted into PHA by some bacterial strains such as *C. necator* [32] and *Pseudomonas chlororaphis* [40]. The use of WCO to produce PHA without any pretreatment steps suggests a suitable route for sustainable production of value-added products.

The total of fish consumption in the globe is about 71 million tons in the year 2020 [54]. A large amount of fish byproducts (20% to 80% of fish weight) is generated from the fishery industries. The WFO content of the fish byproducts is up to 60%, depending on the species and processing procedure [54, 55]. It means that a large amount of WFO is globally available each year. WFO is used as carbon substrate for PHA production by some bacteria such as *Ralstonia* sp. [35], *Salinivibrio* sp. [36], and *Pseudomonas* spp. [41]. In this study, a high yield of PHB was also obtained by *C. necator* by using WFO as sole carbon substrate. This suggests that WFO can be a new efficient carbon substrate for PHA production.

#### 3.1.1 Batch culture for PHB production by *C. necator* H16

**Fig. 4:**
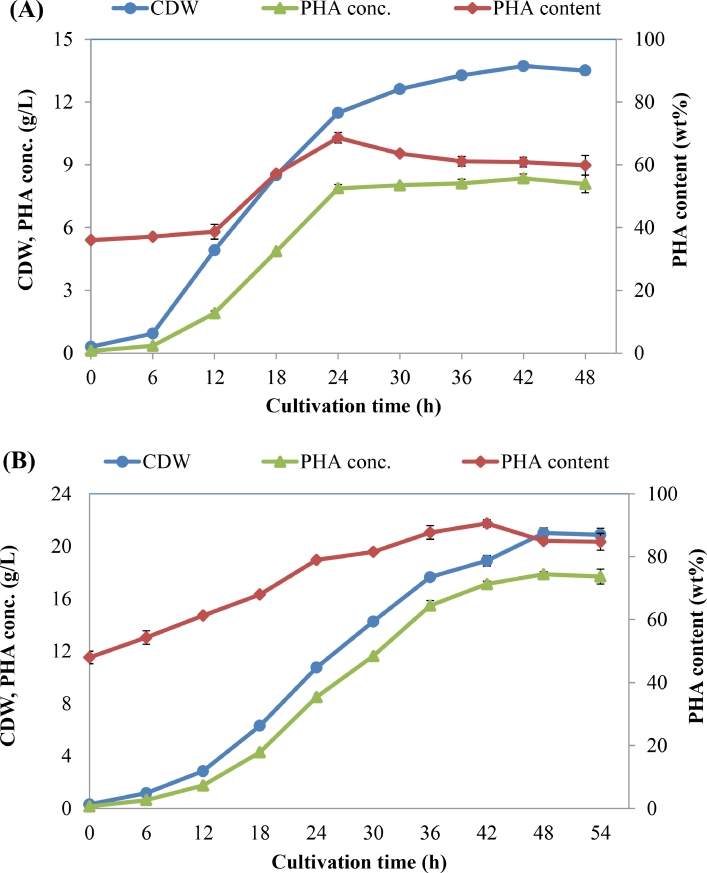
PHB production by *C. necator* H16 from WCO (A) and WFO (B) in batch-culture mode.

The production of PHB by *C. necator* H16 using WCO or WFO as carbon substrate was then investigated by batch cultivation mode in a 3 L bioreactor. Fig. 4A shows the time profile of the growth rate and PHB synthesis by *C. necator* H16 using WCO as the sole carbon source. The CDW was increased during the culture time and reached the highest value of 13.7 g/L at 42 h. The PHB content was also increased during the first 24 h of cultivation to a maximum value of 68.6 wt%, and then reduced after that. The increase in both CDW and PHB contents during the first 24 h of cultivation led to a dramatic increase in PHB concentration, but after that, the PHB content decreased and the CDW still increased. This resulted in a slight increase in PHB concentration to the highest value of 8.4 g/L at 42 h. Maximum PHB content obtained in the bioreactor (68.6 wt%) was much lower than that obtained in the flask (78.5 wt%) from 20 g/L of WCO. As a result, the total cell mass and PHB concentration obtained in the bioreactor were also lower than those obtained in the flask. The results indicate that in the bioreactor, WCO was mainly used for bacterial cell growth and not for PHB synthesis; this is evidenced by the data of residual cell mass obtained in the bioreactor (5.4 g/L at 42 and 48 h), which was higher than that obtained in the flask experiment (4.7 g/L at 48 h).

On the other hand, in the case of WFO, the results show that in the bioreactor, WFO was mainly used for PHB synthesis. Maximum PHB content of up to 90.6 wt% was achieved after 42 h of cultivation. A high CDW of 21 g/L and PHB concentration of 17.9 g/L were obtained after 48 h of cultivation (Fig. 4B). The maximum yield of PHB from WFO was 0.89 g/g, the highest value reported so far when WFO was used as sole carbon source for PHA production. In previous studies, a maximum PHB yield of 0.18 g/g was obtained by *Ralstonia* sp. M91 [35], followed by *Pseudomonas* spp. (less than 0.13 g/g) [41] and *Salinivibrio* sp. M318 (less than 0.1 g/g) [36]. The results obtained in this study indicate that WFO could be an inexpensive and promising carbon substrate for PHA production by *C. necator*, an industrial bacterial species.

#### 3.1.2 Fed-batch culture for PHB production by *C. necator* H16

**Fig. 5:**
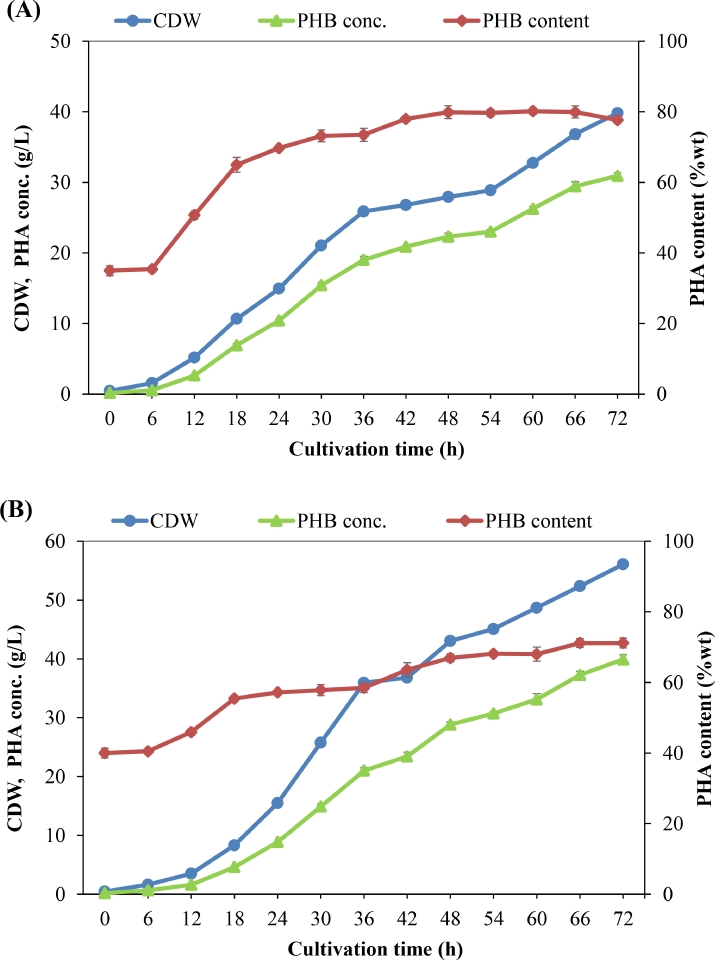
Fed-batch culture profile for PHB production by *C. necator* H16 using mixtures of organic and inorganic nitrogen sources and WCO (A) or WFO (B) as carbon substrate.

In order to increase the cell density and PHB productivity, fed-batch cultivation mode was then applied. The medium (PM1) containing a mixture of organic and inorganic nitrogen sources was first applied. Fig. 5 shows that bacterial cell mass was increased during the cultivation process, and CDWs of 39.8 and 56.1 g/L were reached at 72 h when WCO and WFO were respectively supplied. The content of PHB in bacterial cells was also increased; the highest PHB content of 80.2 wt% was achieved from WCO after 60 h of cultivation, whereas a maximum PHB content of 71.2 wt% was obtained from WFO after 66 h of cultivation. As a result, the concentration of PHB was also increased; the highest values of 30.9 and 39.9 g/L were obtained after 72 h of growing in a medium containing WCO and WFO, respectively. The results of CDW and PHB concentrations obtained in fed-batch fermentation were improved as compared with those obtained in batch fermentation. However, the PHB productivity obtained in the fed-batch fermentation (0.43 g/L/h in the case of WCO and 0.55 g/L/h in the case of WFO) was still very low compared with that obtained by the other studies, e.g., *C. necator* H16 using waste rapeseed oil (1.46 g/L/h) [32] and *C. necator* H16 using palm oil (1.35 g/L/h) [56]. Therefore, further studies need to be performed to improve the PHB productivity. Through comparison of our study with previous studies, we found that the supplemented nitrogen source in the culture medium is a big difference. A mixture of organic and inorganic nitrogen sources (peptone, meat extract, yeast extract, and urea) was used in our study. However, only an inorganic nitrogen source such as (NH_4_)_2_SO_4_ or CO(NH_2_)_2_ was commonly used in other studies for PHA production by *C. necator* [32, 56]. Peptone, meat extract, and yeast extract are complex substrates; besides being nitrogen sources, they can also provide many other essential elements for bacterial growth. The content of nutrients provided by such complex substrates will be difficult to analyze and control during the fermentation process. However, this is not the ideal condition for PHA production because nutrient limitation is well known to influence the synthesis of PHA by most of the PHA producers [57, 58]. For example, previous studies have demonstrated that the C/N ratio is a key factor affecting the accumulation of PHA [59, 60]. Yang et al. [59] found that initial C/N ratios of 20:1 to 40:1 were favorable conditions for growth and PHB accumulation of the bacterial strain *C. necator* H16, but they obtained the highest PHA content of about 70 wt% at 80 C/N ratio. In another study, the effect of four different C:N ratios (5:1, 15:1, 35:1, and 65:1) on PHA accumulation by *Haloferax mediterranei* was also investigated. The highest PHA content of 47.22 wt% was found at a C/N ratio of 35:1, whereas CDW was increased with the increase in nitrogen concentration [60]. For the above reasons, minimum medium with nitrogen limitation is commonly applied for PHA production by *C. necator* and other bacteria [32, 56, [59], [60], [61], [62]].

**Fig. 6:**
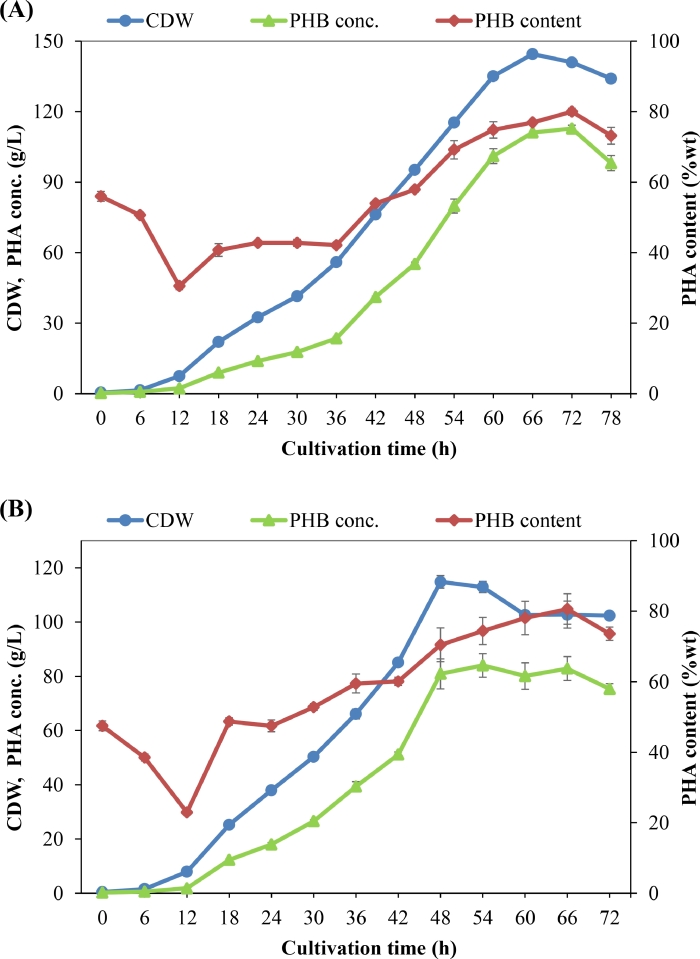
Fed-batch culture profile for PHB production by *C. necator* H16 using urea as nitrogen source and WCO (A) or WFO (B) as carbon substrate.

In this study, a fed-batch fermentation process for PHB production was developed after some trial experiments. The minimum medium containing 2.2 g/L CO(NH_2_)_2_ (PM2) (Table 2) with initial C/N ratio of about 15:1 was applied for PHB production by *C. necator* H16. Different feed solutions were added to the bioreactor during the cultivation process, as described in the Materials and Methods. The added C/N ratio between 15:1 and 20:1 was applied during the first 48 h of cultivation to induce the growth and PHB production of bacterial cells, and nitrogen-free medium was applied after that to induce the accumulation of PHB. As can be seen from Fig. 6, a high CDW of 144.5 g/L was obtained from 150 g/L WCO after 66 h of cultivation, whereas a CDW of 114.8 g/L was obtained from 90 g/L WFO after 48 h of cultivation. The content of PHB in the bacterial cells was high at the beginning of cultivation process because of the limitation of culture conditions in the flasks such as low dissolved oxygen, which induces PHB accumulation in bacterial cells in the seed culture. After transfer to the bioreactor under control conditions, the PHB content decreased during the first 12 h of cultivation and then increased to a maximum value of 82 wt% in the case of WCO at 72 h and 82.6 wt% in the case of WFO at 66 h. A maximum PHB concentration of 115.6 g/L was obtained after 72 h of cultivation when WCO was used as the carbon substrate, 3.75-fold higher than that obtained in the above fed-batch fermentation using PM1 medium (30.9 g/L). In the case of WFO, a maximum PHB concentration of 86.3 g/L was achieved after 54 h of cultivation, 2.17-fold higher than that obtained in the above fed-batch fermentation using PM1 medium (39.9 g/L).

A comparison of PHA production from WCO or WFO in this study with that of the other studies reported so far for plant oil or fish oil in fed-batch cultivation is shown in Table 3. The CDW, PHB content, and PHB concentration obtained in this study from WCO or WFO are comparable to those of the highest reported so far for PHA production from plant oil [31, 32, 56, 63] or fish oil [14]. However, the PHB productivity obtained in this study was 1.74 g/L/h, much higher than that obtained in other studies [31, 32, 36, 40, 56, [63], [64], [65], [66]]. The PHB yield from WCO obtained in this study was 0.8, in the same range with that obtained by strain *C. necator* H16 from waste rapeseed oil (0.83) [32] or obtained by *C. necator* H16 from soybean oil (0.76) [31], but much higher than that obtained in other studies [40, 56, [63], [64], [65], [66]]. This is the first report on PHB production using WFO as sole carbon substrate in fed-batch fermentation, and it is interesting to find that the PHB yield from WFO obtained in this study (0.92 g/g) is among the highest reported so far as compared with those obtained using other carbon substrates. This suggests that WFO is an excellent carbon substrate for PHA production.

**Table 3:** Comparison of PHA production from plant oils or fish oils in fed-batch fermentation by different bacterial strains.

Organism	Carbon source	PHA type	CDW (g/L)	PHA content (wt%)	PHA conc. (g/L)	PHA productivity (g/L/h)	PHA yield (g/g)	Reference
*C. necator* H16	WCO	PHB	135.1	76.9	103.8	1.73	0.8	This work
	WCO	PHB	144.5	78.9	114.1	1.73	0.76	This work
	WFO	PHB	114.8	72.5	83.2	1.73	0.92	This work
*Sanilivibrio* sp. M318	WFO + Glycerol	PHB	69.1	51.5	35.6	0.46	0.32	Thuoc et al. [36]
*C. necator* H16	Waste rapeseed oil	PHB	138	76	105	1.46	0.83	Obruca et al. [32]
*P. chlororaphis* 555	WCO	mcl-PHA^a^	73	19	13.9	0.29	0.11	Ruiz et al. [40]
*C. necator* H16	Palm oil	PHB	156	63	97	1.35	–	Khunthongkaew et al. [56]
*C. necator* H16	Soybean oil	PHB	126	76	95.8	1	0.76	Kahar et al. [31]
*C. necator* PHB-4/pJRDEE32d13	Soybean oil	P(3HB-*co*-3HHx)^b^	138	74	102.1	1.06	0.72	Kahar et al. [31]
*C. necator*								
Re2058/pCB113	Palm oil	P(3HB-*co*-3HHx)^b^	138,8	73	102	1.06	0.63	Riedel et al. [63]
*C. necator* Re2058/pCB113	Sludge palm oil	P(3HB-*co*-3HHx)^b^	88.3	57	50.3	1.1	0.5	Letchimanan et al. [64]
*C. necator* ATCC 17,699	Canola oil	mcl-PHA^a^	20.3	90	18.3	0.45	0.68	López-Cuellar et al. [65]
*C. necator* KCTC 2662	Soybean oil	PHB	32	78	25	0.26	0.42	Park and Kim [66]

a Medium chain length.

b Poly(3-hydroxybutyrate-*co*-3-hydroxyhexanoate).

## 4 Conclusions

The present study showed that high PHB can be produced by the bacterial strain *C. necator* H16 using WCO or WFO as sole carbon substrate. The waste oils can be efficiently converted into a value-added biodegradable product. In addition, WCO, and WFO are lower in price as compared with the other carbon substrates such as sugars and pure oils. The utilization of a low-cost carbon substrate is a promising strategy facilitating an economical PHB production process. Further work on PHB production from WCO and WFO using a larger bioreactor is ongoing.

## Author contributions

Conceptualization, D.V.T. and T.T.L.; investigation, T.T.L., D.T.Q.T., P.Q.H., P.X.N. and D.V.T.; data curation, T.T.L., D.T.Q.T. and D.V.T.; writing—original draft preparation, D.V.T. and T.T.L.; writing-review and editing, D.V.T. All authors have read and agreed to the published version of the manuscript.

## Funding

This research received no external funding.

## Declaration of Competing Interest

All the authors declare that there is no conflict of interest with this study.
